# Impact of dietary supplementation with resistant dextrin (NUTRIOSE^®^) on satiety, glycaemia, and related endpoints, in healthy adults

**DOI:** 10.1007/s00394-021-02618-9

**Published:** 2021-06-25

**Authors:** Mark R. Hobden, Daniel M. Commane, Laetitia Guérin-Deremaux, Daniel Wils, Clementine Thabuis, Agustin Martin-Morales, Saskia Wolfram, Antonio Dìaz, Sineaid Collins, Ines Morais, Ian R. Rowland, Glenn R. Gibson, Orla B. Kennedy

**Affiliations:** 1 Department of Food and Nutritional Sciences, School of Chemistry, Pharmacy and Food, The University of Reading, Reading, UK; 2 Department of Nutrition and Health, Roquette, Lestrem, France; 3 Faculty of Health and Life Sciences, Northumbria University, Newcastle, UK

**Keywords:** Resistant dextrin, Prebiotic, Satiety, Appetite, Glycaemia, Obesity

## Abstract

**Purpose:**

Resistant dextrin (RD) supplementation has been shown to alter satiety, glycaemia, and body weight, in overweight Chinese men; however, there are limited data on its effects in other demographic groups. Here, we investigated the effects of RD on satiety in healthy adults living in the United Kingdom.

**Methods:**

20 normal weight and 16 overweight adults completed this randomised controlled cross-over study. Either RD (14 g/day NUTRIOSE^®^ FB06) or maltodextrin control was consumed in mid-morning and mid-afternoon preload beverages over a 28-day treatment period with crossover after a 28-day washout. During 10-h study visits (on days 1, 14, and 28 of each treatment period), satietogenic, glycaemic and anorectic hormonal responses to provided meals were assessed.

**Results:**

Chronic supplementation with RD was associated with higher fasted satiety scores at day 14 (*P* = 0.006) and day 28 (*P* = 0.040), compared to control. RD also increased satiety after the mid-morning intervention drink, but it was associated with a reduction in post-meal satiety following both the lunch and evening meals (*P* < 0.01). The glycaemic response to the mid-morning intervention drink (0–30 min) was attenuated following RD supplementation (*P* < 0.01). Whilst not a primary endpoint we also observed lower systolic blood pressure at day 14 (*P* = 0.035) and 28 (*P* = 0.030), compared to day 1, following RD supplementation in the normal weight group. Energy intake and anthropometrics were unaffected.

**Conclusions:**

RD supplementation modified satiety and glycaemic responses in this cohort, further studies are required to determine longer-term effects on body weight control and metabolic markers.

**Clinicaltrials.gov registration:**

NCT02041975 (22/01/2014)

**Supplementary Information:**

The online version contains supplementary material available at 10.1007/s00394-021-02618-9.

## Introduction

The relationship between diet, the gut microbiota and aetiology of obesity and metabolic disease is complex [1]. Saccharolytic fermentation of non-digestible carbohydrates (NDC) by the gut microbiota produces short-chain fatty acids (SCFA) [2] that can bind to free fatty acid receptors and stimulate the release of anorectic hormones [3]. There is, therefore, interest in establishing whether increasing dietary intake of NDC, with foods or supplements, modifies satiety responses, and whether when sustained over a period of weeks or months, high intakes of NDC can decrease body weight and metabolic disease risk [4, 5].

Resistant dextrin (RD), a NDC and candidate prebiotic, has been shown to increase the abundance of bacteroides but decrease clostridia in stools from human participants, and importantly, to increase the production of SCFA in both animal and in vitro models [6–8]. In separate 9-week and 12-week dietary intervention studies, RD increased satiety in overweight Chinese men [9], suppressed daily energy intake and reduced body weight [9, 10]. Moreover, Li et al. [11] reported reduced plasma glucose, glycosylated haemoglobin and albumin, lower insulin and an improved homeostatic model assessment of insulin resistance score in an overweight cohort. Elsewhere chronic supplementation with RD improved markers of insulin resistance and inflammation in women with type 2 diabetes [12].

Risk of metabolic disease typically begins at a lower body weight in Asian populations than it does for those of European ancestry [13]. Accordingly, the aim of present study was to investigate the effects of RD supplementation on satiety, ad libitum energy intake, glycaemia, anthropometrics and blood pressure, in normal and overweight adults in the United Kingdom. Here, we describe and discuss findings from a randomised controlled trial (RCT) with a 28-day RD supplementation period with perceptions of satiety over the course of a well-controlled study visit as the primary endpoint.

## Methods

### Study design

The study was approved by the University of Reading Ethics Committee (11/08), carried out in accordance with the Declarations of Helsinki (2008), and registered as a Clinical Trial (NCT02041975). The study was a double-blind, randomised controlled trial, with a cross-over design involving two 28-day treatment periods, consisting of RD or control supplementation, separated by a 28-day washout. Participants attended a familiarisation visit 14 days before the first treatment period and attended study visits on day 1, 14 and 28 of each treatment period during which the satietogenic responses to provided meals over the course of a 10-h study day were assessed.

### Participants

Healthy adults, classified as normal weight or overweight according to accepted BMI classifications [14], were recruited from Berkshire, UK and surrounding areas. Informed written consent was sought from all participants. Inclusion criteria were: aged 22–55 years, normal weight (BMI of 22.0–24.9 kg/m^2^; Asian ethnicity (Chinese or Indian ancestry) 20.0—22.9 kg/m^2^) or overweight (BMI of 25.0–29.9 kg/m^2^; Asian ethnicity 23–26.9 kg/m^2^), resting blood pressure (< 160/90 mmHg), fasted blood haemoglobin (> 115 g/l females, > 125 g/l males), gamma GT (< 80 IU/l), cholesterol (< 6.5 mmol/l), triglycerides (< 1.5 mmol/l) and glucose (< 5.5 mmol/l), non-smoking, weight stable and not looking to lose or gain weight at the time of taking part in the study, cognitive restraint as determined by a three-factor eating questionnaire [15] ≥ 13 and habitual sleep duration > 5 h per night, no known cardiovascular, metabolic or gastrointestinal disease, not pregnant, free from medication (with the exception of contraceptives), no history of drug or alcohol misuse, dietary fibre < 30 g/day (to ensure that with supplement intake, dietary fibre intakes remained less than two SDs above the mean intake in the UK), no antibiotics in the last 6 months, no recent or planned blood donations, not currently using dietary supplements or no willingness to stop their usage for at least 4 weeks prior to, and for the duration of, the study. Participants on the study were reimbursed for their time and expenses. A sample size of 36 was selected based on the findings of Flint et al. [17] who demonstrated that using a study power of 0.8, a difference of 10 mm on fasting, and 5 mm on mean 4.5 h satiety ratings, can be detected with 18 subjects in a paired study design. We, therefore, aimed for 18 overweight and 18 normal weight participants.

### Treatments

Isocaloric orange juice treatment and control beverages were developed containing an equal quantity of orange juice flavouring. The treatment beverages contained 7 g 200 ml^−1^ of resistant dextrin (NUTRIOSE^®^ FB06, Roquette, France) (2 kcal g^−1^), whilst the control drink contained 3.5 g 200 ml^−1^ of maltodextrin (Glucidex) (4 kcal g^−1^). NUTRIOSE® FB06 is a non-viscous RD obtained from wheat with a fibre content of 85% and a mono- and disaccharide content of ≤ 0.5%. Viscosity was similar between both products.

Participants were randomised to treatments with stratification for BMI, gender, age and ethnicity using an Excel-based software package. Investigators and participants were blinded to the treatment arms. Participants were asked to consume 200 ml servings of the study drinks at 10:30 and 15:00 each day totalling 14 g day^−1^ RD, or 7 g day^−1^ of the energy-matched maltodextrin control. (GLUCIDEX^®^21, Roquette, France) (see Table 1 for nutritional information).

**Table 1:** Nutritional information of the RD and control drink

Resistant Dextrin drink		Control drink	
RD (7.0 g NUTRIOSE^®^ FB06, Roquette) and orange juice powder (Clic^®^, Nestlé) mixed with water		Maltodextrin (3.5 g GLUCIDEX®21, Roquette) and orange juice powder (Clic^®^, Nestlé) mixed with water	
Volume	200 ml	Volume	200 ml
Energy	95.9 kcal	Energy	95.9 kcal
Protein	0 g	Protein	0 g
Carbohydrate	22.2 g	Carbohydrate	23.8 g
Sugars	20.4 g	Sugars	20.7 g
Fat	< 0.1 g	Fat	< 0.1 g
Fibre	6 g	Fibre	0 g

Compliance to the dietary intervention, and the effect of the intervention on habitual self-reported food intake were assessed using weighed food diaries; these were completed on the day prior to each of the study visits and at day 7 and day 21 of each arm of the intervention.

### Clinic study visits

Participants were asked to avoid strenuous exercise and alcohol the day before each study visit. Participants consumed a standardised macaroni cheese ready meal as the evening meal the day before each study visit. Males consumed a 500 g meal and females a 400 g meal to reflect gender differences in daily energy intake [16]. Participants arrived for their study visits at 07:45 in a > 12 h fasted state. Fasted body weight and composition measurements were performed after urination and with the subject wearing a disposable gown using a Tanita bioelectrical impedance meter (Tanita ‘BC 418ma’ Analyser, Tanita Inc, USA). Blood pressure was measured, in triplicate, with the subject in supine position (Omron M6 Comfort Monitor, Omron Healthcare, Japan).

### Satiety and ad libitum energy intake

**Fig. 1:**
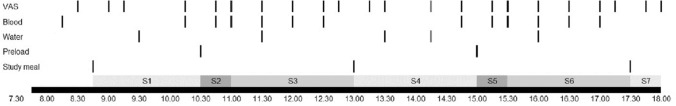
Schematic overview of the study visits. Participants arrived at 07:45 and finished at 18:00. Preloads were provided at 10:30 and 15:00. Standard breakfast, and ad libitum lunch and evening meals were provided at 08:45, 13:00 and 17:30, respectively. Blood samples were collected, VAS questionnaires were completed, and water was provided at the time points shown. For analytical purposes, the study day was divided into time segments (S1–S7)

Perceived appetite was measured using 100 mm VAS at time points shown in Fig. 1 to determine ‘satiety’, ‘hunger’, ‘fullness’, ‘desire-to-eat’ and ‘prospective food consumption’ (PFC) [17]. Appetite VAS questions were anchored at ‘Not at all’ and ‘Extremely’. Participants consumed a gender-specific breakfast, equivalent to 10% of male or female recommended daily energy intakes, as used previously (18), at 08:45. Lunch and evening meals were consumed 150 min after the mid-morning and mid-afternoon ‘preload’ treatment. The lunch meal was a pasta in a tomato sauce, with olive oil and parmesan, and was served ad libitum as described previously [18]. The evening meal of cheese and tomato pizza was also provided ad libitum, with pre-weighed servings provided at 5-min intervals. A VAS questionnaire on palatability and enjoyment was completed exactly 15 min after the start of each study meal and preload.

### Biological sample collection

A qualified healthcare professional inserted a 0.85 mm × 25 mm 23G cannula (Brunz Healthcare, UK) into a vein of the participants’ antecubital fossa at 08:00 and this remained in situ until the last blood sample at 17:00. Blood samples were collected at each study visit at the times shown in Fig. 1. At each time point, 5 ml of venous blood was transferred into a pre-cooled EDTA tube containing 50 μl Inhibitor cocktail (containing DPP-IV, AEBSF and protease cocktail; Merck Millipore, UK) and then stored immediately on ice. Another 3.5 ml of venous blood was transferred into a serum separating tube and kept at room temperature for 30 min to coagulate. All blood samples were centrifuged for 10 min at 1000*g* before separation of plasma/serum and storage at − 80 °C.

### Biological sample analyses

Glucose was analysed using an Instrument Laboratory ILAB 600 auto-analyser (Instrumentation Laboratory Ltd, UK) in serum samples collected at day 1, 14 and 28. Manufacturers’ low and high control standards were used in all batches. Gut hormone concentrations were determined in duplicate using Luminex 200 Technology (Bio-Plex, Bio-Rad, Nazareth, Belgium) and MILLIPLEX MAP Human Metabolic Hormone Magnetic Bead Panel (Millipore, UK) from samples collected at day 1 and 28. The assay was ran as an 8-plex for simultaneous quantification of ghrelin (active), GIP (Total), GLP-1 (Active), insulin, leptin, PYY (Total), interleukin-6 (IL-6) and tumour necrosis factor-α (TNF-α). Blood metabolites were analysed in the morning blood samples (collected between 07:45 and 13:00), and days 1 and 28, only.

### Statistical analysis

All statistical analyses were performed using Predictive Analytics Software version 21.0 for Windows (SPSS Inc., USA). Statistical significance was accepted at the 5% level. The normality of all data was checked using the Shapiro–Wilk test and visually by plotting residuals. Data were analysed for the entire cohort and for the normal weight and overweight sub-groups separately. Correlations between appetite measures were determined using the Pearson correlation coefficient. Logged satiety, metabolite, blood pressure and anthropometric data were analysed using a linear mixed model with repeated measures. Fixed factors in the model were visit (day 1, 14 or 28), treatment (RD or control), and the interaction between visit and treatment (visit*treatment). Where applicable segment was included as a fixed factor. Covariates in the model were treatment order, BMI group, gender, and age, however, these were removed if their effect on the model was non-significant. Fasting satiety/plasma metabolite data and/or energy consumption at lunch or evening meals were used as covariates. Bonferonni correction was used for multiple comparisons. Area under the curve (AUC) values were calculated for satiety and blood metabolite data using the trapezoid rule. AUC values were calculated for key time segments (S): S1, breakfast to morning preload (08:45–10:30); S2, after morning preload (10:30–11:00); S3, after morning preload (11:00–13:00); S4, lunch to afternoon preload (13:00–15:00); S5, post afternoon preload 1 (15:00–15:30); S6, post afternoon preload 2 (15:30–17:30); S7, after evening meal (17:30–18:00). Post preload AUC values were calculated for the first 30 min, to investigate gastric and oro-sensory effects, and from 30 to 150 min, to investigate post-absorptive effects [19].

## Results

Eighty-one adults were screened for the study, 47 met the inclusion criteria and were recruited. Eleven participants dropped out due to illness, work commitments and medication use; however, this was not associated with RD or control supplementation. Of the 36 participants who completed the study, 20 were normal weight and 16 were overweight (see Table 2). Of the 36 participants, 32 (89%) classed themselves as either White-British, White-Irish, or White-European ethnicity, 1 (3%) as Black ethnicity, and 3 (8%) as Asian or Mixed-Asian ethnicity.

**Table 2:** Participant baseline measures

	Normal weight		Overweight	
	(*n* = 20)		(*n* = 16)	
	Male (*n* = 9)	Female (*n* = 11)	Male (*n* = 8)	Female (*n* = 8)
Age (y)	30.8 ± 9.7 (22–50)	31.7 ± 9.0 (22–46)	37.9 ± 9.0 (26–52)	38.0 ± 12.0 (22–55)
Bmi (kg/m^2^)	23.3 ± 1.4 (21.0–24.8)	22.5 ± 1.2 (20.3–24.2)	26.6 ± 1.7 (25.2–29.4)	27.2 ± 0.8 (26.1–28.4)
Body Fat (%)	15.7 ± 4.0 (12.1–23.6)	29.4 ± 3.5 (23.9–35.8)	20.9 ± 5.2 (15.1–29.0)	37.0 ± 2.6 (33.7–41.2)
Waist (cm)	82.2 ± 7.1 (77–100)	74.5 ± 5.1 (65–82)	93.7 ± 9.1 (80–112)	83.9 ± 8.1 (74–97)
Hip (cm)	97.1 ± 3.5 (94–103)	97.9 ± 4.4 (87–104)	104.3 ± 4.8 (97–112)	106.0 ± 3.0 (103–111)
Systolic blood pressure (mmHg)	126.8 ± 7.6 (114.0–136.3)	109.3 ± 19.1 (66.3–126.3)	125.5 ± 6.2 (117.0–133.0)	130.8 ± 19.3 (105.0–160.3)
Diastolic blood pressure (mmHg)	70.8 ± 5.9 (58.7–77.7)	70.7 ± 10.0 (45.3–82.7)	74.5 ± 8.0 (63.3–90.0)	77.4 ± 12.0 (63.0–102.0)
Fasted blood glucose (mmol/l)	4.5 ± 0.3 (4.1–4.9)	4.5 ± 0.2 (4.1–4.8)	4.5 ± 0.3 (4.3–5.0)	4.2 ± 0.4 (3.8–4.9)

Data presented as means ± SD (range)

### Anthropometric, blood pressure and food diary data

**Fig. 2:**
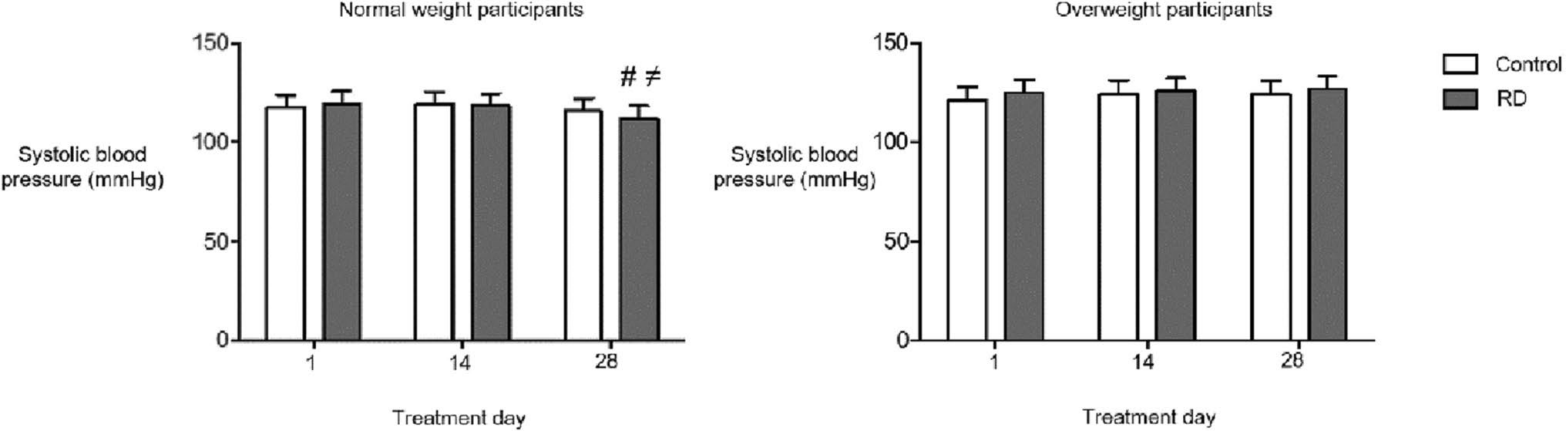
Systolic blood pressure in normal weight and overweight participants following RD and control treatments. ^#^Systolic blood pressure lower at day 28 compared to day 1 (*P* = 0.030) in normal weight participants following RD treatment. ≠ Systolic blood pressure lower at day 28 compared to day 14 (*P* = 0.035) in normal weight participants following RD treatment. Data presented as estimated marginal means ± 95% confidence interval (CI)

Baseline anthropometric and blood pressure data are shown in Table 2. Bodyweight, body fat, waist circumference, hip circumference, and diastolic blood pressure were unaffected by RD or control supplementation. When evaluated across the entire cohort, mean systolic blood pressure was unaltered by RD supplementation. In a sub-analysis by BMI classification, systolic blood pressure was lower at day 28 (mean 112 mmHg) than days 1 (mean 120 mmHg, *P* < 0.030) and 14 (mean 118 mmHg, *P* = 0.035) in normal weight participants consuming RD (Fig. 2). No differences in systolic blood pressure were found after RD supplementation in overweight participants. Neither treatment altered dietary intake at home (assessed using weighed food diaries).

### Satiety ratings

#### Fasting

**Fig. 3:**
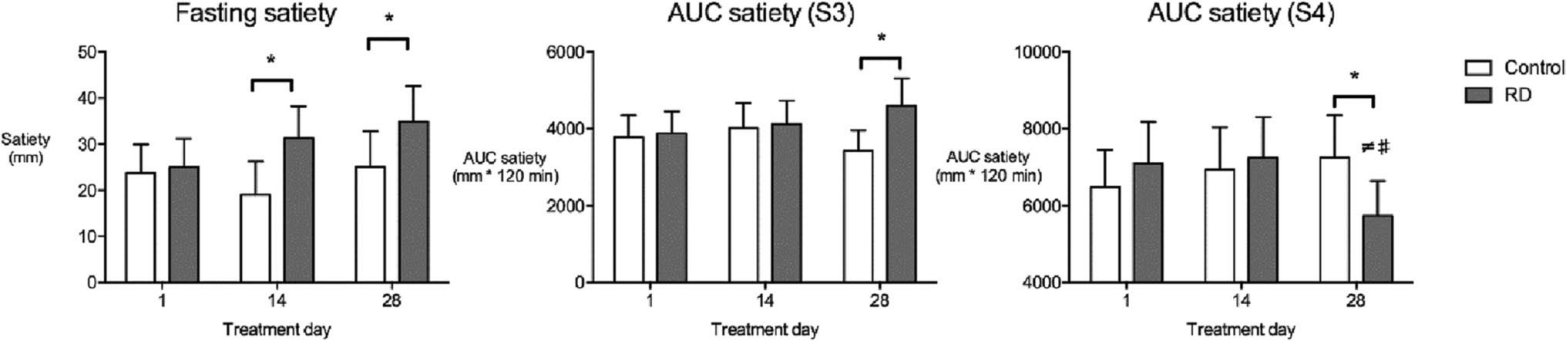
Fasting satiety and AUC satiety for S3 and S4 in the entire cohort. Fasting graph: *Satiety scores higher with RD compared to control at day 14 (*P* = 0.006) and day 28 (*P* = 0.040). AUC satiety S3: * AUC satiety higher with RD compared to control at day 28 (*P* = 0.008). AUC satiety S4: * AUC satiety lower with RD relative to control at day 28 (*P* = 0.004). ≠ AUC satiety lower at day 28 compared to day 1 following RD supplementation (*P* = 0.008). # AUC satiety lower at day 28 compared to day 14 following RD supplementation (*P* = 0.019). Data presented as estimated marginal means ± 95% CI

As shown in Fig. 3 and detailed in Supplementary tables 1–3, fasting satiety scores were significantly higher at day 14 (*P* = 0.006) and day 28 (*P* = 0.040), but not day 1, with RD consumption relative to control. Fasting satiety was higher at day 28 compared to day 1 following RD supplementation both when considered across the entire cohort and in the normal weight group (*P* = 0.012), but not for the overweight group.

#### Postprandial

Postprandial desire-to-eat, hunger and prospective food consumption (all positively correlated, *r* = 0.874*,* P < 0.01) were unaffected by RD supplementation. Postprandial satiety was positively correlated to fullness (*r* 0.841, P < 0.01). Supplementary tables 1, 2 and 3 provide AUC satiety data for S1–S7 for all participants, normal weight participants and overweight participants, respectively. For S1, breakfast to morning preload (08:45–10:30), and S2, after morning preload (10:30–11:00), no treatment or time effects were found for AUC satiety in the entire cohort, or in a sub-analysis by BMI category. For S3, after the morning preload (11:00–13:00) corresponding to the post-breakfast-absorptive phase, the AUC for satiety was higher for RD than for control at day 28 (*P* = 0.008). This difference was found in normal weight participants (*P* = 0.003) but was not observed in overweight participants. For S4, lunch to afternoon preload (13:00–15:00), AUC satiety was significantly lower with RD relative to control at day 28 (*P* = 0.004), and lower at day 28 compared to day 1 (P = 0.019) and day 14 (*P* = 0.008). In a sub-analysis, the same differences were found in normal weight participants (*P* < 0.01) but not in overweight participants. For S5, post afternoon preload 1 (15:00–15:30), no treatment or time effects were found in the AUC for satiety across the cohort; however, a significant difference between treatments at day 28 was found in normal weight (*P* = 0.020) but not in overweight participants. For S6, post afternoon preload 2 (15:30–17:30), no treatment or time effects were found for AUC satiety across the cohort, or in a sub-analysis by BMI category. For S7, after evening meal (17:30–18:00), AUC satiety was lower with RD compared to control at day 28 (*P* = 0.013) and AUC satiety was lower at day 28 compared to day 1 following RD supplementation (*P* = 0.024). Differences were found in the normal weight group but not the overweight group.

### Blood metabolites

#### Fasting

Fasting concentrations of glucose, ghrelin (active), GIP (Total), insulin, leptin and tumour necrosis factor-α (TNF-α) are detailed in Supplementary tables 1–3. Fasting glucose concentrations were unaltered by RD or control supplementation in the entire cohort and normal weight or overweight groups. At day 28, fasting GLP-1 levels were significantly higher following RD compared to control in the entire cohort (*P* < 0.017) and normal weight group (*P* < 0.007); however, no differences were found between day 1 and day 28, suggesting a participant and not a treatment effect. At day 1 (prior to treatment initiation), mean fasting ghrelin was 119.1 in the control arm versus 133.9 in the RD arm (*P* < 0.05).

#### Postprandial

**Fig. 4:**
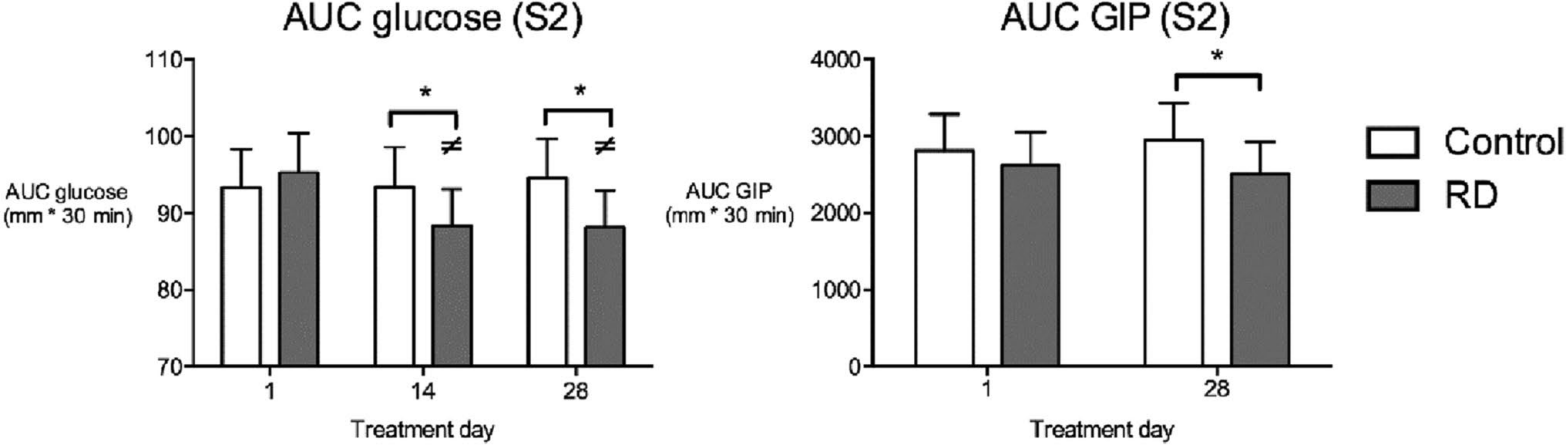
AUC glucose and GIP responses to the morning preload drink in S2 in the entire cohort. * AUC glucose lower with RD than control treatment at day 14 (*P* = 0.044) and day 28 (*P* = 0.009), ≠ AUC glucose lower at day 28 compared to day 1 following RD supplementation (*P* = 0.009) and AUC glucose lower at day 14 compared to day 1 following RD treatment (*P* = 0.009). AUC GIP lower with RD compared to control at day 28 (*P* = 0.002). Data presented as estimated marginal means ± 95% CI

AUC data for S2 and S3 for blood metabolites are provided in supplementary tables 1, 2 and 3. As shown in Fig. 4, postprandial AUC glucose in S2 was lower with RD compared to control at day 28 (*P* = 0.044) in the entire cohort. Moreover, following RD, mean AUC was lower at days 14 and 28, compared to day 1 (P < 0.01). These differences were not found in the overweight group; however in the normal weight group, AUC glucose was lower with RD compared to control at day 28 (*P* = 0.037). AUC GIP for S2 was also lower at day 28 for RD compared to control in the entire cohort (*P* = 0.002) and normal weight (*P* = 0.004), but not overweight, group. Postprandial insulin, TNF-α, GLP-1, and ghrelin responses for S2 were unaltered by RD supplementation in the entire cohort and BMI groups. AUC responses for S3, after morning preload (11:00–13:00), for all blood metabolites were unaffected by RD or control in the entire cohort. AUC GIP was lower following RD compared to control at day 28 in the normal weight group (*P* = 0.011), but not the entire cohort or overweight group. Graphs showing glucose and GIP time-point data are presented as supplementary information (Supplementary Figs. 1 and 2). Interleukin-6 (IL-6) and PYY data were not analysed due to unacceptably high CVs.

### Energy intake

On day 1, the ad libitum energy intake at lunch was higher following the RD mid-morning preload compared to the control drink (mean difference 88 kcal, *P* = 0.028); Supplementary tables 1,2 and 3. This difference was observed in the overweight group (mean difference 170 kcal, (*P* < 0.001) but not in the normal weight group (mean difference 23 kcal, *P* = 0.718). No other treatment nor time effects were observed for ad libitum energy intake at the lunch or evening meals. Mean ratings of enjoyment, taste and palatability of all the study meals and preload drinks were all > 65 (out of 100) and did not differ significantly between treatments or visits.

### Study compliance

No significant differences in self-reported study compliance were found between treatments, with 98% ± 5% (mean ± SD) compliance for control and 98% ± 4% (mean ± SD) compliance reported for RD treatments.

## Discussion

RD supplementation has been shown to alter indices of satiety, glycaemic control, and bodyweight in overweight males in China [9–12], yet there are limited data on the effects of RD consumption on related parameters in other demographic groups, including Western populations. Accordingly, the current study looked to address this knowledge gap. Specifically, we investigated the effects of chronic RD consumption on satiety over the course of in-clinic controlled study visits, in both normal weight and overweight adults living in the UK.

Chronic consumption of RD was compared against an energy-matched maltodextrin control. Habitual diet was unaffected by the intervention, and therefore treatment with the supplementary RD represents a significant increase in fermentable fibre intake over and above control. RD consumption was associated with increased fasting satiety ratings on study visits at day 14 and day 28 of the chronic intervention. In previous work, using an in vitro gut fermentation model, we have shown that RD fermentation elicits increases in short-chain fatty acid concentrations [8]. Of the short-chain fatty acids, propionate in particular is of interest due to its potential anorectic activities [20]; indeed, recent intervention studies have demonstrated that targeted delivery of propionate to the colon in a prebiotic can suppress appetite [21]. We speculate that the chronic supplementation of this fermentable fibre may have increased SCFA production in the gut and, thus, modified perceptions of fasted satiety in our participants. In contrast to previous studies [11, 12], we did not find that either fasting glucose or fasting insulin concentrations were affected by RD supplementation; however, this might be a consequence of our experimental design with a low/normal fasting glucose level used as an inclusion criteria for the study.

RD was also associated with a higher perceived satiety score in the run up to lunch (S3). During the study visits, participants consumed RD, or maltodextrin control, in a drink in the mid-morning period. Postprandial glucose and GIP, an incretin and important marker of nutrient absorption [22], in S2 were lower following the RD drink compared to the control at day 28. The fact that the glycaemic response was significantly lower at days 14 and 28 compared to day 1 suggests that this was a chronic effect of RD supplementation; however, it could also be due to the higher immediate bioavailability of digested sugar from the maltodextrin. Thus, it is possible that the control drink induced a nutrient spike in S2 and a slump in S3, although this was not observed in the metabolite data. This must be considered, alongside higher potential intestinal SCFA production, associated perhaps with RD consumed the previous day, as a potential mechanistic explanation for the higher self-reported feelings of satiety with the RD in the period prior to lunch. This nutrient bioavailaibility hypothesis is consistent with previous acute intervention studies with dietary fibres [23]. It is worth noting that repeated high postprandial glucose responses are associated with progression of diabetes mellitus and cardiovascular diseases due to unfavourable effects on oxidative stress, beta cell function and nutrient transporters [24].

Two and a half hours after the mid-morning preload, the participants consumed lunch; this was followed by a two-hour window until the mid-afternoon preload and then a further two and half hours until the evening meal. At day 28, the RD was associated with reduced satiety post- both the lunch and evening meals relative to control. There was no difference in calorie intake at either meal to explain this. Unfortunately, blood metabolite data from the morning, including appetite related hormones, did not provide a possible explanation for these responses. The morning preload will not have reached the colon by lunch but may well have arrived in the caecum by the time of the evening meal; in vitro batch culture fermentation of prebiotics suggests that their utilisation in the gut takes place over many hours [25]. It is plausible that the concentration of circulating short-chain fatty acids is high following the overnight fast because the microbiota were still utilising the previous days RD, and that the SCFA concentrations began to decline, due to substrate depletion, through the study day. Further, the more immediate availability of energy from the maltodextrin control in the morning and afternoon preloads may have had carry-over effects on nutrient processing following the lunch and evening meals.

In a sub-analysis by BMI category, we noted that the effects of RD supplementation on satiety were found exclusively amongst the normal weight adults. Previous research has shown that the satiety response correlates with the dose of RD consumed [9]. The 14 g/day RD provided in the current study may have been insufficient to alter satiety responses in the overweight adults. There is also evidence that the microbial community of individuals with a high body weight might have a different capacity for the utilisation of non-digestible carbohydrate [26]. Future studies should explore the differential production of short-chain fatty acids in response to prebiotics in body weight associated enterotypes.

Whilst not an a priori defined endpoint for this study, we noted that RD consumption was associated with a clinically meaningful reduction in systolic blood pressure, albeit only in the normal weight participants. This observation has not previously been reported for RD but it is a purported benefit of other NDC [27], and even small reductions in systolic blood pressure are associated with reduced risk of coronary heart disease, coronary artery disease and stroke [28]; future intervention studies should evaluate this further.

Limitations to the current study: First, whilst the energy content of the drinks were matched, total carbohydrate and sugar content of the RD and control drinks were marginally different (Table 1). Nonetheless, glycaemic responses to RD and control morning preloads were comparable at the start of the treatment periods (day 1). Second, the length of the treatment period (28 days) may have been too short to observe any anthropometric changes. We only observed statistically significant responses to the intervention in our normal weight participants; there were, however, more of these (*n* = 20), than the overweight participants (*n* = 16). It may be important to include a larger sample of overweight participants in follow up work.

In conclusion, this study provides evidence that chronic consumption of RD reduces postprandial glycaemia and influences fasted satiety in healthy adults living in the UK. Intervention studies of a greater duration are needed to determine whether RD consumption can help regulate body weight and influence metabolic health. From a mechanistic perspective studies of the differential impact of RD on the microbiota of normal and overweight individuals might help to explain our findings. Finally, the observed potential anti-hypertensive properties of RD are of interest but require further investigation.

## Supplementary Information

Below is the link to the electronic supplementary material.

Supplementary figure 1: Mean plasma glucose concentrations at each time point over the morning section of the clinic visits at days 1, 14 and 28 of each treatment period

Supplementary figure 2: Mean plasma GIP concentrations at each time point over the morning section of the clinic visits at days 1 and 28 of each treatment period

Supplementary figure 3: Mean satiety ratings at each time point over the morning and afternoon sections of the clinic visits at days 1, 14 and 28 of each treatment period

Supplementary file4 (DOCX 77 kb)

## Abbreviations

RD: Resistant dextrin
RCT: Randomised controlled trial
S1-S7: Time segments
NDC: Non-digestible carbohydrate
UK: United Kingdom
VAS: Visual analogue scales
