# Correction to: Impact of dietary supplementation with resistant dextrin (NUTRIOSE®) on satiety, glycaemia, and related endpoints, in healthy adults

**DOI:** 10.1007/s00394-021-02663-4

**Published:** 2021-08-25

**Authors:** Mark R. Hobden, Daniel M. Commane, Laetitia Guérin-Deremaux, Daniel Wils, Clementine Thabuis, Agustin Martin-Morales, Saskia Wolfram, Antonio Dìaz, Sineaid Collins, Ines Morais, Ian R. Rowland, Glenn R. Gibson, Orla B. Kennedy

**Affiliations:** 1 Department of Food and Nutritional Sciences, School of Chemistry, Pharmacy and Food, The University of Reading, Reading, UK; 2 Department of Nutrition and Health, Roquette, Lestrem, France; 3 Faculty of Health and Life Sciences, Northumbria University, Newcastle, UK

## Correction to: European Journal of Nutrition 10.1007/s00394-021-02618-9

The original version of this article unfortunately contained a mistake. In abstracts section, Clinicaltrials.gov registration should be NCT02041975 (22/01/2014).

The original article has been corrected.

