# Supplementary figures and images for: Impact of dietary supplementation with resistant dextrin (NUTRIOSE®) on satiety, glycaemia, and related endpoints, in healthy adults
Source: Eur J Nutr. 2021 Jun 25;60(8):4635–43. doi: 10.1007/s00394-021-02618-9 (PMC8572182; doi:10.1007/s00394-021-02618-9)

Supplementary Figure 1

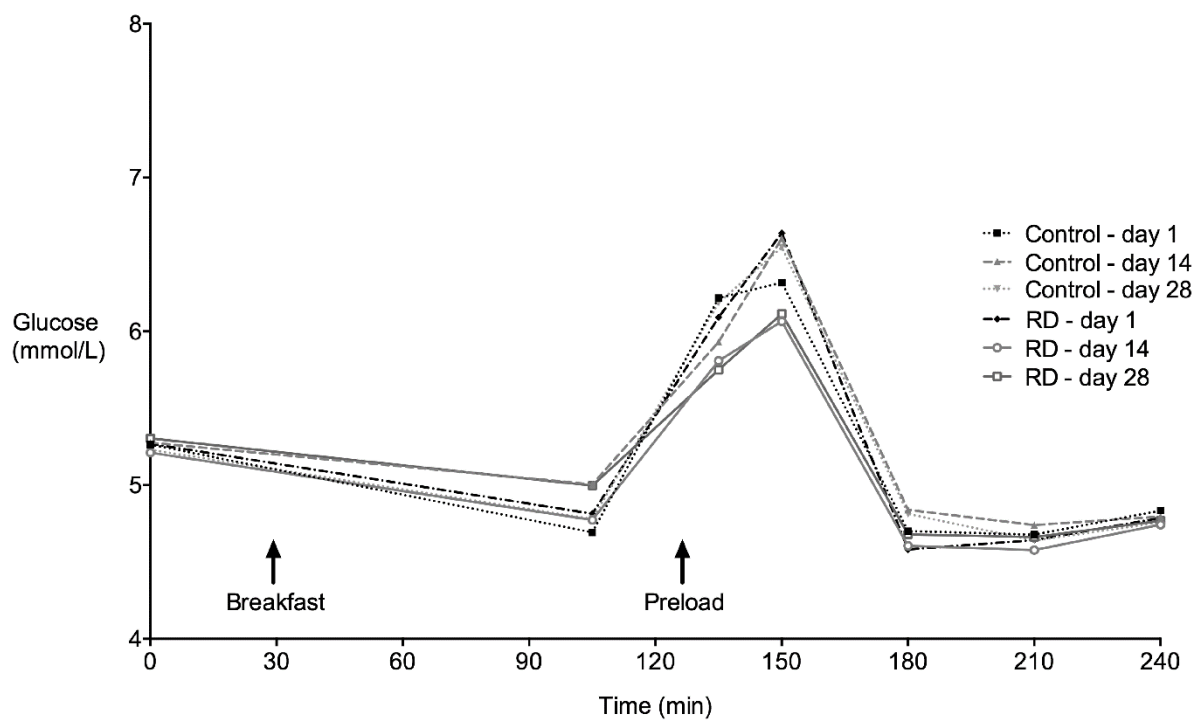

Supplement: Supplementary file 1 — Supplementary figure 1: Mean plasma glucose concentrations at each time point over the morning section of the clinic visits at days 1, 14 and 28 of each treatment period [file 394_2021_2618_MOESM1_ESM.pdf]

Supplementary figure 2

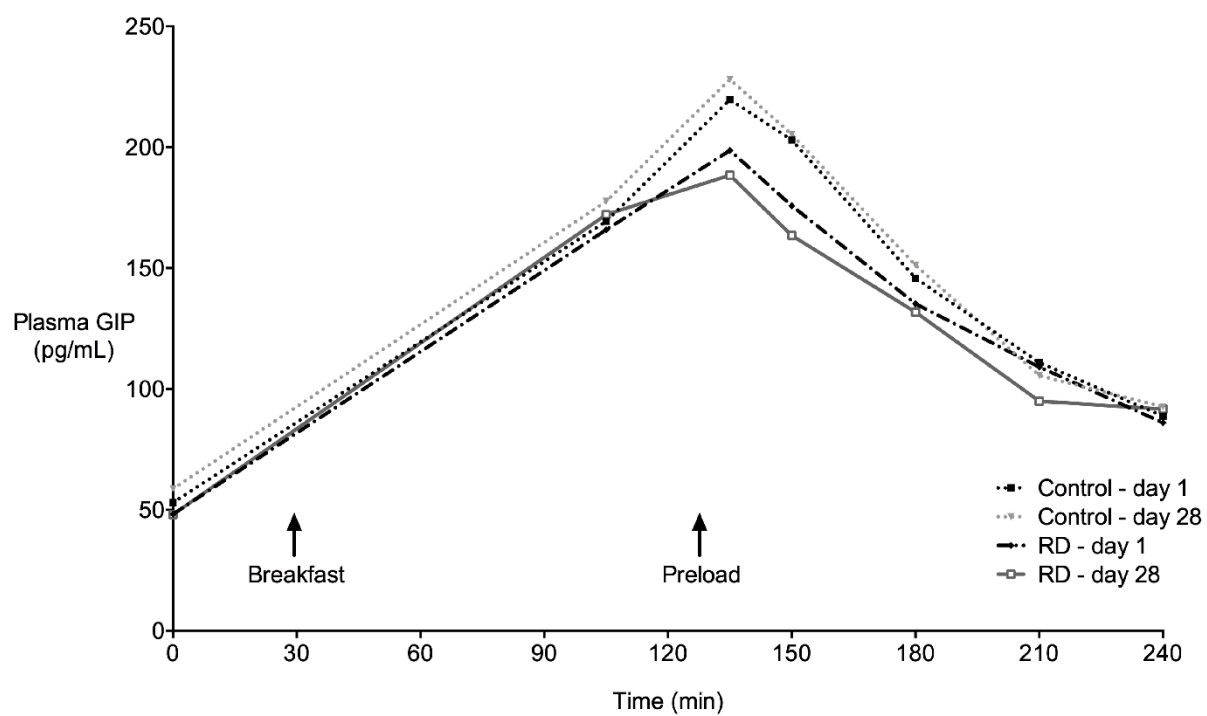

Supplement: Supplementary file 2 — Supplementary figure 2: Mean plasma GIP concentrations at each time point over the morning section of the clinic visits at days 1 and 28 of each treatment period [file 394_2021_2618_MOESM2_ESM.pdf]

Supplementary Figure 3

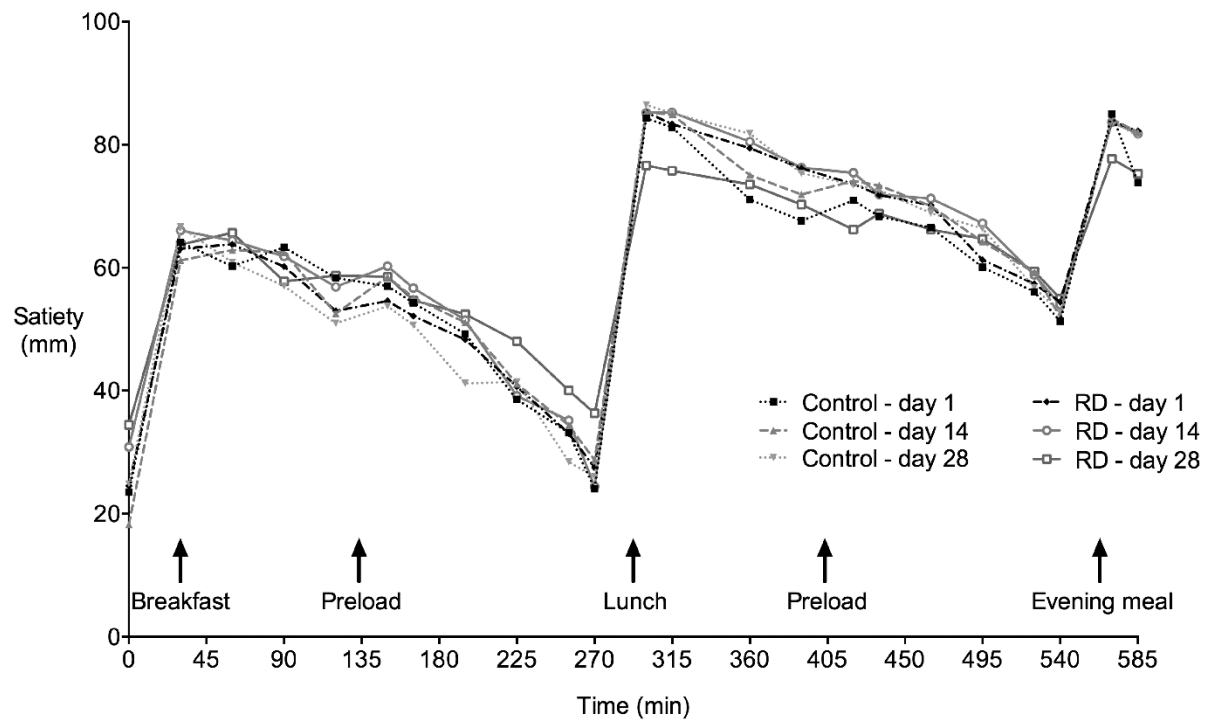

Supplement: Supplementary file 3 — Supplementary figure 3: Mean satiety ratings at each time point over the morning and afternoon sections of the clinic visits at days 1, 14 and 28 of each treatment period [file 394_2021_2618_MOESM3_ESM.pdf]
