# Supplementary material for: Impact of dietary supplementation with resistant dextrin (NUTRIOSE®) on satiety, glycaemia, and related endpoints, in healthy adults
Source: Eur J Nutr. 2021 Jun 25;60(8):4635–43. doi: 10.1007/s00394-021-02618-9 (PMC8572182; doi:10.1007/s00394-021-02618-9)
Supplement: Supplementary file 4 — Supplementary file4 (DOCX 77 kb) [file 394_2021_2618_MOESM4_ESM.docx]

**Supplementary table 1** Linear mixed model outcomes for the primary study endpoints for the complete cohort (n =36)

|  |  | **All participants** | | | | | | | | | | | | | | | | | |  |
| --- | --- | --- | --- | --- | --- | --- | --- | --- | --- | --- | --- | --- | --- | --- | --- | --- | --- | --- | --- | --- |
|  |  | **Control** | | | | | | | | | **Resistant Dextrin** | | | | | | | | |  |
|  |  | Day 1 | | | Day 14 | | | Day 28 | | | Day 1 | | | Day 14 | | | Day 28 | | | *Significant differences* |
| **Fasting** |  |  |  |  |  |  |  |  |  |  |  |  |  |  |  |  |  |  |  |  |
| Satiety | *mm* | 17 | ± | 4 | 14 | ± | 4 ^t^ | 19 | ± | 5 ^t*^ | 21 | ± | 5 | 22 | ± | 5 ^t^ | 27 | ± | 7 ^t*^ | t (P=0.006), t* (P=0.040) |
| Glucose | *mmol/l* | 5.2 | ± | 0.1 | 5.2 | ± | 0.1 | 5.2 | ± | 0.1 | 5.2 | ± | 0.1 | 5.2 | ± | 0.1 | 5.2 | ± | 0.1 | none |
| Insulin | *pg/ml* | 229.8 | ± | 75.9 |  |  |  | 223.4 | ± | 74.0 | 177.9 | ± | 59.0 |  |  |  | 205.2 | ± | 67.8 | none |
| GIP | *pg/ml* | 44.6 | ± | 7.7 |  |  |  | 48.6 | ± | 8.5 | 43.1 | ± | 7.5 |  |  |  | 43.6 | ± | 7.5 | none |
| GLP-1 | *pg/ml* | 1.5 | ± | 0.5 |  |  |  | 1.1 | ± | 0.4 ^t^ | 1.7 | ± | 0.6 |  |  |  | 1.8 | ± | 0.7 ^t^ | t (P=0.024) |
| Ghrelin | *pg/ml* | 119.1 | ± | 22.7 ^t^ |  |  |  | 124.5 | ± | 23.6 | 133.9 | ± | 25.4 ^t^ |  |  |  | 127.1 | ± | 24.2 | t (P=0.026) |
| Leptin | *pg/ml* | 4179.7 | ± | 1213.6 |  |  |  | 4346.0 | ± | 1265.0 | 4614.7 | ± | 1339.9 |  |  |  | 4385.2 | ± | 1273.3 | none |
| TNF-α | *pg/ml* | 2.8 | ± | 0.5 |  |  |  | 2.9 | ± | 0.5 | 3.0 | ± | 0.5 |  |  |  | 3.2 | ± | 0.6 | none |
|  |  |  |  |  |  |  |  |  |  |  |  |  |  |  |  |  |  |  |  |  |
| **S1** (before morning drink) |  |  |  |  |  |  |  |  |  |  |  |  |  |  |  |  |  |  |  |  |
| Satiety | *mm * 105 min* | 5234 | ± | 684 | 5229 | ± | 710 | 4910 | ± | 654 | 5182 | ± | 686 | 5271 | ± | 684 | 5193 | ± | 696 | none |
|  |  |  |  |  |  |  |  |  |  |  |  |  |  |  |  |  |  |  |  |  |
| **S2** (after morning drink) |  |  |  |  |  |  |  |  |  |  |  |  |  |  |  |  |  |  |  |  |
| Satiety | *mm * 30 min* | 749 | ± | 98 | 804 | ± | 109 | 709 | ± | 94 | 708 | ± | 94 | 839 | ± | 109 | 780 | ± | 105 | none |
| Glucose | *mmol/l * 30 min* | 93.3 | ± | 4.7 | 93.4 | ± | 5.0 ^t^ | 94.5 | ± | 4.9 ^t*^ | 95.3 | ± | 4.9 ^d^ | 88.3 | ± | 4.6 ^t,^ ^d, d*^ | 88.1 | ± | 4.6 ^t*, d*^ | d (P=0.009), d* (P=0.009), t (P=0.044), t* (P=0.009) |
| Insulin | *pg/ml * 30 min* | 17120.0 | ± | 2633.1 |  |  |  | 17051.6 | ± | 2433.7 | 17309.3 | ± | 2691.4 |  |  |  | 15978.5 | ± | 2307.9 | none |
| GIP | *pg/ml * 30 min* | 2813.0 | ± | 406.3 |  |  |  | 2945.4 | ± | 420.4 ^t^ | 2609.7 | ± | 377.0 |  |  |  | 2520.0 | ± | 357.5 ^t^ | t (P=0.002) |
| GLP-1 | *pg/ml * 30 min* | 44.1 | ± | 17.8 |  |  |  | 59.6 | ± | 22.7 | 71.8 | ± | 29.1 |  |  |  | 65.2 | ± | 25.0 | none |
| Ghrelin | *pg/ml * 30 min* | 1280.5 | ± | 186.1 |  |  |  | 1306.4 | ± | 169.5 | 1224.1 | ± | 178.9 |  |  |  | 1348.8 | ± | 179.7 | none |
| Leptin | *pg/ml * 30 min* | 58454.3 | ± | 2961.9 ^t^ |  |  |  | 55603.4 | ± | 3185.7 | 53960.1 | ± | 2785.4 ^t^ |  |  |  | 54502.4 | ± | 3276.5 | t (P=0.020) |
| TNF-α | *pg/ml * 30 min* | 43.6 | ± | 3.4 |  |  |  | 41.2 | ± | 1.8 | 39.6 | ± | 3.1 |  |  |  | 41.7 | ± | 2.0 | none |
|  |  |  |  |  |  |  |  |  |  |  |  |  |  |  |  |  |  |  |  |  |
| **S3** (before lunch) |  |  |  |  |  |  |  |  |  |  |  |  |  |  |  |  |  |  |  |  |
| Satiety | *mm * 120 min* | 3809 | ± | 498 | 3984 | ± | 541 | 3419 | ± | 453 ^t^ | 3878 | ± | 513 | 4134 | ± | 533 | 4398 | ± | 590 ^t^ | t (P=0.008) |
| Glucose | *mmol/l * 120 min* | 445.4 | ± | 18.7 | 455.8 | ± | 20.1 | 448.1 | ± | 19.3 | 451.2 | ± | 19.4 | 441.0 | ± | 19.0 | 442.3 | ± | 19.0 | none |
| Insulin | *pg/ml * 120 min* | 44756.9 | ± | 6579.4 |  |  |  | 45935.8 | ± | 6438.0 | 44756.9 | ± | 6464.7 |  |  |  | 46397.5 | ± | 6422.8 | none |
| GIP | *pg/ml * 120 min* | 11192.5 | ± | 1711.9 |  |  |  | 11014.9 | ± | 1619.2 | 10270.2 | ± | 1562.2 |  |  |  | 9936.8 | ± | 1443.8 | none |
| GLP-1 | *pg/ml * 120 min* | 175.9 | ± | 76.0 |  |  |  | 274.8 | ± | 102.4 | 283.4 | ± | 120.1 |  |  |  | 285.1 | ± | 105.0 | none |
| Ghrelin | *pg/ml * 120 min* | 10026.6 | ± | 826.3 |  |  |  | 11070.1 | ± | 1103.4 | 9946.7 | ± | 801.4 |  |  |  | 10764.4 | ± | 1053.5 | none |
| Leptin | *pg/ml * 120 min* | 327747.9 | ± | 17849.4 |  |  |  | 316791.8 | ± | 22596.2 | 318061.5 | ± | 17021.0 |  |  |  | 323838.4 | ± | 23098.8 | none |
| TNF-α | *pg/ml * 120 min* | 267.5 | ± | 14.3 |  |  |  | 248.9 | ± | 16.6 | 263.7 | ± | 13.4 |  |  |  | 268.5 | ± | 17.9 | none |
|  |  |  |  |  |  |  |  |  |  |  |  |  |  |  |  |  |  |  |  |  |
| **Lunch energy intake** | *kcal* | 697 | ± | 89 ^t^ | 711 | ± | 92 | 784 | ± | 100 | 798 | ± | 101 ^t^ | 734 | ± | 93 | 788 | ± | 100 | t (P=0.028) |
|  |  |  |  |  |  |  |  |  |  |  |  |  |  |  |  |  |  |  |  |  |
| **S4** (after lunch) |  |  |  |  |  |  |  |  |  |  |  |  |  |  |  |  |  |  |  |  |
| Satiety | *mm * 120 min* | 6529 | ± | 853 | 6877 | ± | 934 | 7223 | ± | 956 ^t^ | 7115 | ± | 942 ^d^ | 7259 | ± | 936 ^d*^ | 5497 | ± | 737 ^t, d, d*^ | t (P=0.004), d (P=0.019), d* (P=0.008) |
|  |  |  |  |  |  |  |  |  |  |  |  |  |  |  |  |  |  |  |  |  |
| **S5** (after afternoon drink) |  |  |  |  |  |  |  |  |  |  |  |  |  |  |  |  |  |  |  |  |
| Satiety | *mm * 30 min* | 986 | ± | 130 | 1073 | ± | 146 | 1034 | ± | 137 | 1039 | ± | 138 | 1056 | ± | 137 | 896 | ± | 122 | none |
|  |  |  |  |  |  |  |  |  |  |  |  |  |  |  |  |  |  |  |  |  |
| **S6** (before evening meal) |  |  |  |  |  |  |  |  |  |  |  |  |  |  |  |  |  |  |  |  |
| Satiety | *mm * 120 min* | 5955 | ± | 783 | 6516 | ± | 885 | 6419 | ± | 850 | 6292 | ± | 833 | 6674 | ± | 866 | 6039 | ± | 810 | none |
|  |  |  |  |  |  |  |  |  |  |  |  |  |  |  |  |  |  |  |  |  |
| **Evening meal energy intake** | *kcal* | 520 | ± | 76 | 539 | ± | 80 | 532 | ± | 78 | 527 | ± | 77 | 557 | ± | 81 | 500 | ± | 73 | none |
|  |  |  |  |  |  |  |  |  |  |  |  |  |  |  |  |  |  |  |  |  |
| **S7** (after evening meal) |  |  |  |  |  |  |  |  |  |  |  |  |  |  |  |  |  |  |  |  |
| Satiety | *mm * 30 min* | 1162 | ± | 152 | 1213 | ± | 165 | 1193 | ± | 158 ^t^ | 1212 | ± | 160 ^d^ | 1143 | ± | 147 | 944 | ± | 127 ^t, d^ | t (P=0.013), d (P=0.024) |
|  |  |  |  |  |  |  |  |  |  |  |  |  |  |  |  |  |  |  |  |  |
| Data presented as estimated marginal means ± 95% CI | | |  |  |  |  |  |  |  |  |  |  |  |  |  |  |  |  |  |  |
| ^t^ indicates significant difference between treatments | | |  |  |  |  |  |  |  |  |  |  |  |  |  |  |  |  |  |  |
| ^d^ indicates significant difference between days | |  |  |  |  |  |  |  |  |  |  |  |  |  |  |  |  |  |  |  |

**Supplementary table 2** Linear mixed model outcomes for the primary study endpoints for the normal weight participants (n =20)

|  |  | **Normal weight participants** | | | | | | | | | | | | | | | | | | |
| --- | --- | --- | --- | --- | --- | --- | --- | --- | --- | --- | --- | --- | --- | --- | --- | --- | --- | --- | --- | --- |
|  |  | **Control** | | | | | | |  |  | **Resistant Dextrin** | | | | | | | | |  |
|  |  | Day 1 |  |  | Day 14 |  |  | Day 28 |  |  | Day 1 |  |  | Day 14 |  |  | Day 28 |  |  | *Significant differences* |
| **Fasting** |  |  |  |  |  |  |  |  |  |  |  |  |  |  |  |  |  |  |  |  |
| Satiety | *mm* | 15 | ± | 5 | 15 | ± | 5 | 21 | ± | 7 | 16 | ± | 5 ^d^ | 21 | ± | 7 | 30 | ± | 10 ^d^ | d (P=0.012) |
| Glucose | *mmol/l* | 5.1 | ± | 0.1 | 5.1 | ± | 0.1 | 5.0 | ± | 0.1 | 5.1 | ± | 0.1 | 5.0 | ± | 0.1 | 5.1 | ± | 0.1 | none |
| Insulin | *pg/ml* | 233.2 | ± | 93.0 ^t^ |  |  |  | 225.0 | ± | 90.4 | 127.9 | ± | 51.0 ^t^ |  |  |  | 167.8 | ± | 67.0 | t (P=0.002) |
| GIP | *pg/ml* | 43.0 | ± | 10.5 |  |  |  | 50.9 | ± | 12.5 | 39.4 | ± | 9.6 |  |  |  | 41.7 | ± | 10.1 | none |
| GLP-1 | *pg/ml* | 1.7 | ± | 0.9 |  |  |  | 1.0 | ± | 0.5 ^t^ | 2.0 | ± | 1.0 |  |  |  | 2.0 | ± | 1.0 ^t^ | t (P=0.033) |
| Ghrelin | *pg/ml* | 115.8 | ± | 26.7 ^t^ |  |  |  | 123.5 | ± | 28.6 | 146.2 | ± | 33.7 ^t^ |  |  |  | 136.9 | ± | 31.5 | t (P=0.001) |
| Leptin | *pg/ml* | 3371.1 | ± | 1072.6 |  |  |  | 3491.2 | ± | 1115.6 | 3944.2 | ± | 1255.0 |  |  |  | 3435.8 | ± | 1093.2 | none |
| TNF-α | *pg/ml* | 2.5 | ± | 0.7 |  |  |  | 2.5 | ± | 0.7 | 2.8 | ± | 0.8 |  |  |  | 3.0 | ± | 0.8 | none |
|  |  |  |  |  |  |  |  |  |  |  |  |  |  |  |  |  |  |  |  |  |
| **S1** (before morning drink) | |  |  |  |  |  |  |  |  |  |  |  |  |  |  |  |  |  |  |  |
| Satiety | *mm * 105 min* | 4934 | ± | 899 | 4866 | ± | 926 | 4633 | ± | 881 | 5019 | ± | 934 | 5319 | ± | 968 | 5049 | ± | 936 | none |
|  |  |  |  |  |  |  |  |  |  |  |  |  |  |  |  |  |  |  |  |  |
| **S2** (after morning drink) | |  |  |  |  |  |  |  |  |  |  |  |  |  |  |  |  |  |  |  |
| Satiety | *mm * 30 min* | 745 | ± | 136 | 769 | ± | 146 | 665 | ± | 127 | 668 | ± | 124 | 786 | ± | 142 | 757 | ± | 141 | none |
| Glucose | *mmol/l * 30 min* | 89.6 | ± | 6.0 | 90.7 | ± | 6.2 | 92.2 | ± | 6.3 ^t^ | 92.5 | ± | 6.1 | 85.6 | ± | 5.8 | 85.7 | ± | 5.7 ^t^ | t (P=0.037) |
| Insulin | *pg/ml * 30 min* | 15867.1 | ± | 3082.4 |  |  |  | 16680.6 | ± | 3010.0 | 16203.8 | ± | 3173.9 |  |  |  | 15444.4 | ± | 2761.6 | none |
| GIP | *pg/ml * 30 min* | 2681.1 | ± | 523.0 |  |  |  | 2921.9 | ± | 562.9 ^t^ | 2591.5 | ± | 501.4 |  |  |  | 2418.7 | ± | 458.1 ^t^ | t (P=0.004) |
| GLP-1 | *pg/ml * 30 min* | 58.5 | ± | 29.7 |  |  |  | 71.8 | ± | 34.9 | 80.6 | ± | 40.7 |  |  |  | 67.7 | ± | 32.2 | none |
| Ghrelin | *pg/ml * 30 min* | 1449.5 | ± | 282.8 |  |  |  | 1468.5 | ± | 260.2 | 1261.4 | ± | 245.1 |  |  |  | 1366.5 | ± | 237.6 | none |
| Leptin | *pg/ml * 30 min* | 57182.3 | ± | 3812.5 |  |  |  | 56331.0 | ± | 4226.8 | 52785.9 | ± | 3420.8 |  |  |  | 57988.5 | ± | 4190.0 | none |
| TNF-α | *pg/ml * 30 min* | 44.7 | ± | 4.7 |  |  |  | 40.6 | ± | 2.4 | 38.7 | ± | 3.9 |  |  |  | 42.1 | ± | 2.4 | none |
|  |  |  |  |  |  |  |  |  |  |  |  |  |  |  |  |  |  |  |  |  |
| **S3** (before lunch) | |  |  |  |  |  |  |  |  |  |  |  |  |  |  |  |  |  |  |  |
| Satiety | *mm * 120 min* | 3425 | ± | 624 | 3530 | ± | 671 | 3069 | ± | 586 ^t^ | 3771 | ± | 702 | 3874 | ± | 705 | 4741 | ± | 879 ^t^ | t (P=0.003) |
| Glucose | *mmol/l * 120 min* | 432.7 | ± | 23.6 | 437.5 | ± | 24.7 | 436.2 | ± | 25.0 | 432.2 | ± | 23.5 | 416.1 | ± | 23.1 | 424.1 | ± | 23.5 | none |
| Insulin | *pg/ml * 120 min* | 43001.9 | ± | 7935.5 |  |  |  | 47762.7 | ± | 8422.6 | 44267.2 | ± | 8060.5 |  |  |  | 46910.6 | ± | 8233.7 | none |
| GIP | *pg/ml * 120 min* | 10894.4 | ± | 2229.8 |  |  |  | 11684.4 | ± | 2307.4 ^t^ | 10219.0 | ± | 2059.0 |  |  |  | 9710.9 | ± | 1886.5 ^t^ | t (P=0.011) |
| GLP-1 | *pg/ml * 120 min* | 192.9 | ± | 102.8 ^t^ |  |  |  | 311.1 | ± | 147.7 | 427.9 | ± | 223.4 ^t^ |  |  |  | 372.4 | ± | 173.7 | t (P=0.042) |
| Ghrelin | *pg/ml * 120 min* | 10883.5 | ± | 1201.7 |  |  |  | 11602.8 | ± | 1515.8 | 9946.7 | ± | 1062.8 |  |  |  | 10646.6 | ± | 1363.1 | none |
| Leptin | *pg/ml * 120 min* | 322223.3 | ± | 23282.7 |  |  |  | 322223.3 | ± | 29495.0 | 311451.8 | ± | 20474.7 |  |  |  | 336717.6 | ± | 29902.7 | none |
| TNF-α | *pg/ml * 120 min* | 269.1 | ± | 18.7 |  |  |  | 250.9 | ± | 22.3 | 261.4 | ± | 16.9 |  |  |  | 271.8 | ± | 23.4 | none |
|  |  |  |  |  |  |  |  |  |  |  |  |  |  |  |  |  |  |  |  |  |
| **Lunch energy intake** | *kcal* | 733 | ± | 118 | 692 | ± | 112 | 803 | ± | 129 | 761 | ± | 122 | 677 | ± | 109 | 786 | ± | 126 | none |
|  |  |  |  |  |  |  |  |  |  |  |  |  |  |  |  |  |  |  |  |  |
| **S4** (after lunch) | |  |  |  |  |  |  |  |  |  |  |  |  |  |  |  |  |  |  |  |
| Satiety | *mm * 120 min* | 6562 | ± | 1195 | 6775 | ± | 1289 | 7563 | ± | 1439 ^t^ | 7252 | ± | 1344 ^d^ | 7266 | ± | 1317 ^d*^ | 4447 | ± | 828 ^t, d, d*^ | t (P<0.001), d (P=0.002), d* (P=0.002) |
|  |  |  |  |  |  |  |  |  |  |  |  |  |  |  |  |  |  |  |  |  |
| **S5** (after afternoon drink) | |  |  |  |  |  |  |  |  |  |  |  |  |  |  |  |  |  |  |  |
| Satiety | *mm * 30 min* | 1042 | ± | 190 | 1085 | ± | 206 | 1123 | ± | 214 ^t^ | 1073 | ± | 199 | 1038 | ± | 189 | 800 | ± | 151 ^t^ | t (P=0.020) |
|  |  |  |  |  |  |  |  |  |  |  |  |  |  |  |  |  |  |  |  |  |
| **S6** (before evening meal) | |  |  |  |  |  |  |  |  |  |  |  |  |  |  |  |  |  |  |  |
| Satiety | *mm * 120 min* | 6535 | ± | 1190 | 6568 | ± | 1249 | 6836 | ± | 1300 | 6292 | ± | 1166 | 6503 | ± | 1184 | 5739 | ± | 1068 | none |
|  |  |  |  |  |  |  |  |  |  |  |  |  |  |  |  |  |  |  |  |  |
| **Evening meal energy intake** | *kcal* | 523 | ± | 104 | 538 | ± | 108 | 556 | ± | 111 | 559 | ± | 111 | 548 | ± | 110 | 478 | ± | 95 | none |
|  |  |  |  |  |  |  |  |  |  |  |  |  |  |  |  |  |  |  |  |  |
| **S7** (after evening meal) | |  |  |  |  |  |  |  |  |  |  |  |  |  |  |  |  |  |  |  |
| Satiety | *mm * 30 min* | 1192 | ± | 217 | 1180 | ± | 224 | 1208 | ± | 230 ^t^ | 1264 | ± | 235 ^d^ | 1074 | ± | 196 ^d*^ | 739 | ± | 138 ^t, d, d*^ | t (P<0.001), d (P<0.001), d* (P=0.024) |
|  |  |  |  |  |  |  |  |  |  |  |  |  |  |  |  |  |  |  |  |  |
| Data presented as estimated marginal means ± 95% CI | | | |  |  |  |  |  |  |  |  |  |  |  |  |  |  |  |  |  |
| ^t^ indicates significant difference between treatments | | | |  |  |  |  |  |  |  |  |  |  |  |  |  |  |  |  |  |
| ^d^ indicates significant difference between days | | | |  |  |  |  |  |  |  |  |  |  |  |  |  |  |  |  |  |

**Supplementary table 3** Linear mixed model outcomes for the primary study endpoints for the overweight participants (n =16)

|  |  | **Overweight participants** | | | | | | | | | | | | | | | | | |  |
| --- | --- | --- | --- | --- | --- | --- | --- | --- | --- | --- | --- | --- | --- | --- | --- | --- | --- | --- | --- | --- |
|  |  | **Control** | | | | | | | | | **Resistant Dextrin** | | | | | | | | |  |
|  |  | Day 1 | | | Day 14 | | | Day 28 | | | Day 1 | | | Day 14 | | | Day 28 | | | *Significant differences* |
| **Fasting** |  |  |  |  |  |  |  |  |  |  |  |  |  |  |  |  |  |  |  |  |
| Satiety | *mm* | 19 | ± | 6 | 12 | ± | 4 ^t^ | 17 | ± | 6 | 30 | ± | 10 | 24 | ± | 8 ^t^ | 23 | ± | 8 | t (P=0.008) |
| Glucose | *mmol/l* | 5.4 | ± | 0.2 | 5.4 | ± | 0.2 | 5.4 | ± | 0.2 | 5.4 | ± | 0.2 | 5.4 | ± | 0.2 | 5.4 | ± | 0.2 | none |
| Insulin | *pg/ml* | 225.0 | ± | 106.7 |  |  |  | 223.6 | ± | 106.1 | 283.7 | ± | 134.7 |  |  |  | 268.3 | ± | 127.1 | none |
| GIP | *pg/ml* | 46.7 | ± | 10.7 |  |  |  | 46.1 | ± | 10.5 | 49.1 | ± | 11.5 |  |  |  | 46.3 | ± | 10.6 | none |
| GLP-1 | *pg/ml* | 1.2 | ± | 0.5 |  |  |  | 1.2 | ± | 0.6 | 1.5 | ± | 0.7 |  |  |  | 1.5 | ± | 0.7 | none |
| Ghrelin | *pg/ml* | 123.3 | ± | 41.4 |  |  |  | 125.5 | ± | 42.0 | 119.1 | ± | 40.0 |  |  |  | 115.5 | ± | 38.8 | none |
| Leptin | *pg/ml* | 5574.7 | ± | 2605.7 |  |  |  | 5808.0 | ± | 2717.8 | 5630.8 | ± | 2637.9 |  |  |  | 6063.2 | ± | 2834.0 | none |
| TNF-α | *pg/ml* | 3.1 | ± | 0.6 |  |  |  | 3.5 | ± | 0.6 | 3.2 | ± | 0.6 |  |  |  | 3.5 | ± | 0.6 | none |
|  |  |  |  |  |  |  |  |  |  |  |  |  |  |  |  |  |  |  |  |  |
| **S1** (before morning drink) | |  |  |  |  |  |  |  |  |  |  |  |  |  |  |  |  |  |  |  |
| Satiety | *mm * 105 min* | 5676 | ± | 973 | 5722 | ± | 1000 | 5229 | ± | 879 | 5443 | ± | 933 | 5213 | ± | 881 | 5362 | ± | 937 | none |
|  |  |  |  |  |  |  |  |  |  |  |  |  |  |  |  |  |  |  |  |  |
| **S2** (after morning drink) | |  |  |  |  |  |  |  |  |  |  |  |  |  |  |  |  |  |  |  |
| Satiety | *mm * 30 min* | 757 | ± | 130 | 849 | ± | 149 | 761 | ± | 128 | 769 | ± | 132 | 909 | ± | 153 | 807 | ± | 141 | none |
| Glucose | *mmol/l * 30 min* | 97.2 | ± | 7.3 | 96.1 | ± | 7.7 | 96.8 | ± | 7.3 | 98.1 | ± | 7.5 | 91.2 | ± | 7.0 | 90.6 | ± | 7.0 | none |
| Insulin | *pg/ml * 30 min* | 18471.8 | ± | 4229.1 |  |  |  | 17430.9 | ± | 3733.0 | 18490.3 | ± | 4304.4 |  |  |  | 16531.1 | ± | 3605.1 | none |
| GIP | *pg/ml * 30 min* | 2951.3 | ± | 618.2 |  |  |  | 2969.1 | ± | 612.4 | 2628.1 | ± | 552.5 |  |  |  | 2628.1 | ± | 548.3 | none |
| GLP-1 | *pg/ml * 30 min* | 33.3 | ± | 18.0 |  |  |  | 49.5 | ± | 25.2 | 64.0 | ± | 35.1 |  |  |  | 62.9 | ± | 33.1 | none |
| Ghrelin | *pg/ml * 30 min* | 1130.0 | ± | 237.6 |  |  |  | 1162.1 | ± | 220.1 | 1189.2 | ± | 255.6 |  |  |  | 1331.4 | ± | 268.3 | none |
| Leptin | *pg/ml * 30 min* | 59694.8 | ± | 4644.6 |  |  |  | 54830.4 | ± | 4719.2 | 55160.4 | ± | 4444.2 |  |  |  | 51225.9 | ± | 4967.4 | none |
| TNF-α | *pg/ml * 30 min* | 42.5 | ± | 4.9 |  |  |  | 41.8 | ± | 2.8 | 40.6 | ± | 4.8 |  |  |  | 41.2 | ± | 3.0 | none |
|  |  |  |  |  |  |  |  |  |  |  |  |  |  |  |  |  |  |  |  |  |
| **S3** (before lunch) | |  |  |  |  |  |  |  |  |  |  |  |  |  |  |  |  |  |  |  |
| Satiety | *mm * 120 min* | 4398 | ± | 757 | 4647 | ± | 816 | 3858 | ± | 652 | 4044 | ± | 693 | 4492 | ± | 755 | 3948 | ± | 693 | none |
| Glucose | *mmol/l * 120 min* | 458.1 | ± | 28.4 | 474.9 | ± | 31.2 | 459.9 | ± | 28.5 | 470.6 | ± | 30.1 | 467.3 | ± | 29.8 | 461.3 | ± | 29.5 | none |
| Insulin | *pg/ml * 120 min* | 46583.4 | ± | 10340.5 |  |  |  | 44178.8 | ± | 9252.3 | 45297.2 | ± | 9913.7 |  |  |  | 45935.8 | ± | 9620.3 | none |
| GIP | *pg/ml * 120 min* | 11498.8 | ± | 2561.4 |  |  |  | 10383.8 | ± | 2199.3 | 10321.7 | ± | 2283.1 |  |  |  | 10157.8 | ± | 2143.4 | none |
| GLP-1 | *pg/ml * 120 min* | 160.6 | ± | 92.7 |  |  |  | 243.0 | ± | 122.1 | 187.9 | ± | 107.1 |  |  |  | 218.5 | ± | 109.6 | none |
| Ghrelin | *pg/ml * 120 min* | 9228.0 | ± | 1124.9 |  |  |  | 10561.8 | ± | 1552.6 | 9936.8 | ± | 1228.8 |  |  |  | 10894.4 | ± | 1601.5 | none |
| Leptin | *pg/ml * 120 min* | 333700.8 | ± | 29026.1 |  |  |  | 311451.8 | ± | 33834.2 | 324486.8 | ± | 27334.5 |  |  |  | 311140.5 | ± | 33800.4 | none |
| TNF-α | *pg/ml * 120 min* | 265.6 | ± | 21.4 |  |  |  | 247.2 | ± | 24.6 | 265.9 | ± | 20.7 |  |  |  | 265.3 | ± | 26.4 | none |
|  |  |  |  |  |  |  |  |  |  |  |  |  |  |  |  |  |  |  |  |  |
| **Lunch energy intake** | *kcal* | 640 | ± | 128 ^t^ | 713 | ± | 146 | 743 | ± | 149 | 867 | ± | 173 ^t^ | 832 | ± | 167 | 808 | ± | 161 | t (P=0.001) |
|  |  |  |  |  |  |  |  |  |  |  |  |  |  |  |  |  |  |  |  |  |
| **S4** (after lunch) | |  |  |  |  |  |  |  |  |  |  |  |  |  |  |  |  |  |  |  |
| Satiety | *mm * 120 min* | 6496 | ± | 1113 | 7009 | ± | 1230 | 6850 | ± | 1151 | 7002 | ± | 1206 | 7252 | ± | 1225 | 7303 | ± | 1282 | none |
|  |  |  |  |  |  |  |  |  |  |  |  |  |  |  |  |  |  |  |  |  |
| **S5** (after afternoon drink) | |  |  |  |  |  |  |  |  |  |  |  |  |  |  |  |  |  |  |  |
| Satiety | *mm * 30 min* | 918 | ± | 157 | 1055 | ± | 184 | 939 | ± | 158 | 1004 | ± | 172 | 1078 | ± | 181 | 1030 | ± | 181 | none |
|  |  |  |  |  |  |  |  |  |  |  |  |  |  |  |  |  |  |  |  |  |
| **S6** (before evening meal) | |  |  |  |  |  |  |  |  |  |  |  |  |  |  |  |  |  |  |  |
| Satiety | *mm * 120 min* | 5266 | ± | 902 | 6438 | ± | 1125 | 5979 | ± | 1010 | 6336 | ± | 1091 | 6891 | ± | 1164 | 6445 | ± | 1131 | none |
|  |  |  |  |  |  |  |  |  |  |  |  |  |  |  |  |  |  |  |  |  |
| **Evening meal energy intake** | *kcal* | 518 | ± | 114 | 541 | ± | 122 | 505 | ± | 111 | 490 | ± | 108 | 568 | ± | 126 | 527 | ± | 116 | none |
|  |  |  |  |  |  |  |  |  |  |  |  |  |  |  |  |  |  |  |  |  |
| **S7** (after evening meal) | |  |  |  |  |  |  |  |  |  |  |  |  |  |  |  |  |  |  |  |
| Satiety | *mm * 30 min* | 1127 | ± | 193 | 1255 | ± | 220 | 1174 | ± | 198 | 1157 | ± | 198 | 1235 | ± | 209 | 1309 | ± | 230 | none |
|  |  |  |  |  |  |  |  |  |  |  |  |  |  |  |  |  |  |  |  |  |
| Data presented as estimated marginal means ± 95% CI | | | |  |  |  |  |  |  |  |  |  |  |  |  |  |  |  |  |  |
| ^t^ indicates significant difference between treatments | | | |  |  |  |  |  |  |  |  |  |  |  |  |  |  |  |  |  |
| ^d^ indicates significant difference between days | | | |  |  |  |  |  |  |  |  |  |  |  |  |  |  |  |  |  |
